# Correlation of Ankle-Brachial Pressure Index (ABPI) and Clinical Findings With Duplex Scan for the Assessment of Peripheral Arterial Disease

**DOI:** 10.7759/cureus.112148

**Published:** 2026-07-06

**Authors:** Md. Rajib Mahmud, Golam Mahmud, SK Muhammad Atiqur Rahman, A H M Masiur Rahman, Nadia Hossain, G. M Alomgir Kabir, H. M Tareque, Mehedi Hasan, Al Amin Rahman

**Affiliations:** 1 Spine Surgery, National Institute of Traumatology and Orthopaedic Rehabilitation, Dhaka, BGD; 2 Orthopedics, National Institute of Traumatology and Orthopaedic Rehabilitation, Dhaka, BGD; 3 Neonatology, Dhaka Medical College Hospital, Dhaka, BGD; 4 Orthopedic Surgery, National Institute of Traumatology and Orthopaedic Rehabilitation, Dhaka, BGD; 5 Surgery, Ibn Sina Specialized Hospital, Dhaka, BGD

**Keywords:** ankle-brachial pressure index, atherosclerosis, duplex ultrasonography, intermittent claudication, lower extremity ischemia, peripheral arterial disease (pad), vascular screening

## Abstract

Background and objective

Peripheral arterial disease (PAD) is a common manifestation of systemic atherosclerosis and a powerful predictor of coronary and cerebrovascular events, yet it is frequently underdiagnosed, particularly in primary care and resource-limited settings. The ankle-brachial pressure index (ABPI) is an inexpensive, noninvasive, first-line test, whereas arterial duplex ultrasonography provides anatomical and hemodynamic information that can guide revascularization. The objective of this study was to correlate the ABPI and clinical findings with arterial duplex scan findings in the evaluation of lower-limb PAD.

Materials and methods

This descriptive cross-sectional study enrolled 50 patients with clinically suspected chronic lower-limb arterial occlusive disease admitted to the Department of Surgery, Dhaka Medical College Hospital, between September 2018 and March 2019. All patients underwent history taking, clinical examination, ABPI measurement, and arterial duplex ultrasonography of the affected limb. An ABPI of 0.90 or less, supported by clinical findings, was considered positive for PAD, and stenosis or occlusion on duplex was taken as the imaging finding of interest. Agreement between ABPI plus clinical findings and duplex was assessed, and the corresponding diagnostic performance was calculated. Analyses were performed using IBM SPSS Statistics for Windows, version 20.0 (released 2011; IBM Corp., Armonk, NY, USA), with P < 0.05 considered statistically significant.

Results

The mean age was 53.4 ± 11.5 years, and 82.0% of patients were male (male-to-female ratio, 4.5:1). Smoking (88.0%) was the most frequent risk factor, followed by diabetes mellitus (64.0%), hypertension (58.0%), and obesity (44.0%). Intermittent claudication and muscle cramping (68.0% each) were the most common symptoms, and all patients had tissue loss (gangrene or ulceration). ABPI indicated moderate disease (0.41-0.70) in 38.0% of patients, mild disease (0.71-0.90) in 28.0%, and severe disease (<0.40) in 16.0%. Duplex demonstrated stenosis or occlusion in 76.0% of patients. ABPI combined with clinical findings showed a sensitivity of 90.2%, specificity of 88.9%, positive predictive value of 97.3%, and overall accuracy of 90.0%, with a statistically significant association with duplex findings (P = 0.0001).

Conclusions

ABPI combined with clinical assessment correlated strongly with duplex ultrasonography and provided an accurate, accessible approach to the diagnosis of lower-limb PAD. The combined use of physiological and imaging data supports earlier diagnosis and rational treatment planning, an advantage that is especially relevant in resource-limited settings.

## Introduction

Peripheral arterial disease (PAD) encompasses a group of disorders that produce progressive stenosis, occlusion, or aneurysmal dilation of the aorta and its noncoronary branches, most commonly through atherosclerosis [1]. In the lower limb, the clinical consequences of PAD, such as intermittent claudication, ischemic rest pain, tissue loss, and amputation, reduce functional capacity and quality of life and impose a substantial personal, social, and economic burden. Because PAD reflects systemic atherosclerosis, it is also a powerful marker of coronary and cerebrovascular disease and is associated with an increased risk of myocardial infarction, ischemic stroke, and death [1,2].

The epidemiology of PAD is closely tied to conventional atherosclerotic risk factors. The prevalence of disease rises with age, reaching 12-20% in individuals older than 65 years, although a large proportion of affected people remain asymptomatic [3,4]. Tobacco use is the single most important modifiable risk factor; smokers are two to three times more likely to develop lower-limb PAD than nonsmokers, and the risk increases with the number of cigarettes smoked and the duration of use [2,5]. Diabetes mellitus increases the risk of PAD approximately two- to fourfold through endothelial and smooth muscle dysfunction, in proportion to the severity and duration of disease [6]. Other established risk factors include older age, male sex, hypertension, dyslipidemia, and a family history of vascular disease [7-12]. Elevated total and low-density lipoprotein cholesterol levels and reduced high-density lipoprotein cholesterol levels have each been correlated with accelerated disease, with the risk increasing by roughly 5-10% for every 10 mg/dL increase in total cholesterol [11,13-15].

Several diagnostic tools are available for PAD, and they can usually be matched to the clinical presentation in an efficient, stepwise manner. When PAD is suspected, the first-line test is the ankle-brachial pressure index (ABPI), which is noninvasive, inexpensive, and reproducible. An ABPI of 0.90 or less establishes the diagnosis and also carries prognostic value for limb survival, wound healing, and overall survival [1,16]. When the ABPI is abnormal, arterial duplex ultrasonography is generally the next step, providing both anatomical localization and hemodynamic characterization of stenoses; contrast angiography, computed tomographic angiography, and magnetic resonance angiography are reserved for selected cases or preprocedural planning [17-19]. The additional information from duplex scanning can determine whether disease is amenable to endovascular or surgical intervention and can guide the technical approach.

Despite its accuracy, the ABPI has been incompletely adopted in primary care, partly because of the time required and the need for a rest period before measurement [1,20]. In resource-limited settings such as Bangladesh, patients frequently present late with established tissue loss, and a pragmatic strategy that combines clinical assessment, ABPI, and selective duplex imaging is therefore particularly attractive [21,22]. We conducted this study to correlate the ABPI and clinical findings with arterial duplex ultrasonography in patients with lower-limb PAD, with the aim of clarifying how well a clinically driven, ABPI-based assessment agrees with duplex findings in this population.

## Materials and methods

Study design, setting, and participants

This was a prospective, observational, cross-sectional (noninterventional) study conducted in the Department of Surgery, Dhaka Medical College Hospital, Dhaka, Bangladesh, over a six-month period from September 16, 2018, to March 15, 2019. Each enrolled patient was assessed at a single time point (cross-sectional evaluation), with prospective, consecutive recruitment of eligible admissions. A total of 50 patients who satisfied the eligibility criteria were included.

Patients were enrolled by purposive sampling of consecutive admissions who met the eligibility criteria, reflecting the clinical reality of a tertiary surgical ward in which patients typically present with established disease. The inclusion criteria were (1) clinical symptoms and signs of chronic arterial occlusive disease of the lower limbs and (2) provision of informed written consent. The exclusion criteria were malignancy, jaundice, pregnancy, renal failure, ongoing chemotherapy or radiotherapy, clinical features of varicose veins, and arterial disease confined to the coronary, aortic arch, or cerebral territories. These exclusions were applied to remove conditions that could confound the interpretation of the ABPI or duplex study or that represent nonatherosclerotic or venous pathology. For example, renal failure is associated with medial arterial calcification and noncompressible vessels, varicose veins reflect venous rather than arterial disease, and pregnancy and malignancy introduce distinct hemodynamic and safety considerations. Each eligible patient underwent a standardized sequence of history taking, general and local examination, baseline investigations, ABPI measurement, and arterial duplex ultrasonography, with findings recorded prospectively on a prestructured case record form.

Sample size

The sample size was estimated using the following standard formula:

$n = \frac{z^{2}pq}{d^{2}},$

where z = 1.96, p = 0.13 (the estimated prevalence of symptomatic and asymptomatic lower-limb PAD in adults older than 50 years) [3], q = 1 − p = 0.87, and d = 0.10. This yielded a minimum required sample of 43; given the study duration, 50 patients were enrolled.

Operational definitions and measurements

PAD was defined as obstruction of the large arteries of the lower limb outside the coronary, aortic arch, and cerebral territories, most often due to atherosclerosis. The ABPI was defined as the ratio of the higher systolic pressure at the ankle (dorsalis pedis or posterior tibial artery) to the higher brachial systolic pressure, measured by handheld Doppler after the patient had rested in the supine position for 10 minutes. Following established guideline thresholds, ABPI values were categorized as normal (1.00-1.29), borderline (0.91-0.99), mild disease (0.71-0.90), moderate disease (0.41-0.70), and severe disease (<0.40); a value greater than 1.40 was considered indicative of a noncompressible vessel [1,3]. Arterial duplex ultrasonography of the affected limb was performed in all patients, with assessment of the flow pattern (monophasic versus triphasic), spectral characteristics, and the presence of stenosis or occlusion. Baseline investigations included a complete blood count, serum bilirubin, blood glucose, serum creatinine, and chest radiography. No proprietary or copyrighted scoring instrument was used in this study. The ABPI is a quantitative pressure ratio, and Buerger’s test is a clinical examination maneuver. Disease severity was graded using the published guideline ABPI thresholds cited above; therefore, no licensing or permission was required.

Data collection and statistical analysis

Data were recorded on a prestructured case record form by the study physician and were checked for consistency before analysis. Continuous variables are presented as mean ± SD, and categorical variables as frequencies and percentages. Agreement between ABPI plus clinical findings and duplex ultrasonography was examined using a two-by-two table. Sensitivity, specificity, positive and negative predictive values, and overall accuracy were calculated, with 95% CIs derived using the Wilson score method. The association between the two methods was tested using Fisher’s exact test, which was chosen because of the small expected cell counts. Analyses were performed using IBM SPSS Statistics for Windows, version 20.0 (released 2011; IBM Corp., Armonk, NY, USA), and a two-sided P-value <0.05 was considered statistically significant.

Ethical considerations

The research protocol was approved by the Ethical Committee of Dhaka Medical College. The aims, procedures, risks, and benefits of the study were explained to each participant in an easily understandable local language, and informed written consent was obtained before enrollment. Participants were assured of confidentiality, and the study was conducted in accordance with the principles of the Declaration of Helsinki.

## Results

**Figure 1:**
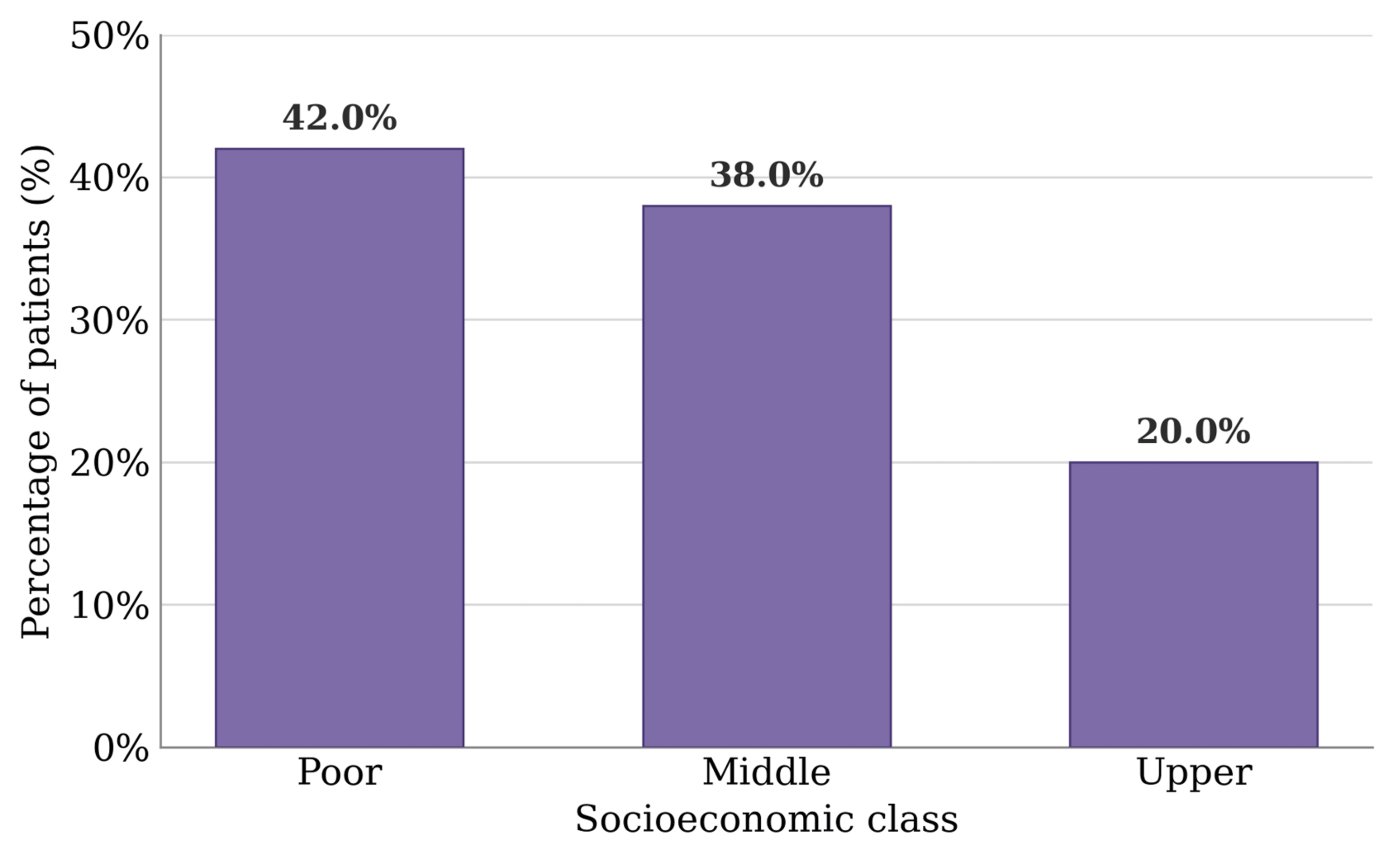
Distribution of the study patients by socioeconomic class (n = 50) The horizontal axis (x-axis) shows socioeconomic class, and the vertical axis (y-axis) shows the percentage of patients. The values correspond to those presented in Table 1 (poor, 42.0%; middle, 38.0%; upper, 20.0%).

Fifty patients with clinically suspected lower-limb PAD were studied. The mean age was 53.4 ± 11.5 years, with the highest incidence in the sixth decade (58.0%), followed by the fifth decade (30.0%). Forty-one patients (82.0%) were male, and nine (18.0%) were female, giving a male-to-female ratio of 4.5:1. Two-thirds of patients (66.0%) were from urban areas, and most belonged to the poor (42.0%) or middle (38.0%) socioeconomic classes (Figure 1). The demographic and baseline characteristics are summarized in Table 1.

**Table 1:** Demographic and baseline characteristics of the patients (n = 50) Data are presented as n (%) unless otherwise indicated.

Characteristic	Value, n (%)
Age group, years	
30-40	4 (8.0)
41-50	15 (30.0)
51-60	29 (58.0)
>60	2 (4.0)
Mean ± SD age, years	53.4 ± 11.5
Sex	
Male	41 (82.0)
Female	9 (18.0)
Male-to-female ratio	4.5:1
Residence	
Urban	33 (66.0)
Rural	17 (34.0)
Socioeconomic status	
Poor	21 (42.0)
Middle	19 (38.0)
Upper	10 (20.0)
Occupation	
Day laborer	10 (20.0)
Business	10 (20.0)
Housewife	9 (18.0)
Service holder	8 (16.0)
Retired	7 (14.0)
Farmer	6 (12.0)

Smoking was the most common risk factor, present in 88.0% of patients, followed by diabetes mellitus (64.0%), hypertension (58.0%), and obesity (44.0%); concomitant heart disease (26.0%) and hyperlipidemia (10.0%) were less frequent (Table 2).

**Table 2:** Distribution of major cardiovascular risk factors (n = 50) Data are presented as n (%).

Risk factor	n (%)
Smoking	44 (88.0)
Diabetes mellitus	32 (64.0)
Hypertension	29 (58.0)
Obesity	22 (44.0)
Heart disease	13 (26.0)
Hyperlipidemia	5 (10.0)

Regarding symptoms, intermittent claudication and muscle cramping were the most common, each reported by 68.0% of patients, followed by numbness (56.0%), rest pain (18.0%), and dull ache (14.0%). The duration of symptoms ranged from approximately one month to 1.4 years, and nearly two-thirds of patients (62.0%) sought care more than six months after symptom onset. On examination, gangrene or foot ulceration was present in all patients, with the toes being the most common ulcer site (62.0%). Other frequent findings included pallor with paresthesia (64.0%), prolonged capillary refill (54.0%), skin coolness (52.0%), and increased local temperature (48.0%) (Table 3).

**Table 3:** Clinical symptoms, examination findings, and ulcer location (n = 50) Data are presented as n (%); more than one clinical feature could be present in a given patient.

Clinical feature	n (%)
Symptoms	
Intermittent claudication	34 (68.0)
Muscle cramping	34 (68.0)
Numbness	28 (56.0)
Rest pain	9 (18.0)
Dull ache	7 (14.0)
Examination findings	
Gangrene or foot ulceration	50 (100.0)
Pallor with paresthesia	32 (64.0)
Prolonged capillary refill	27 (54.0)
Skin coolness	26 (52.0)
Increased local temperature	24 (48.0)
Skin color change	17 (34.0)
Positive Buerger’s test	15 (30.0)
Hair loss	13 (26.0)
Ulcer location	
Toes	31 (62.0)
Foot	12 (24.0)
Heel	5 (10.0)
Leg	2 (4.0)

Assessment of peripheral pulses showed that the dorsalis pedis, anterior tibial, and posterior tibial arteries were most frequently affected, whereas femoral involvement was uncommon. The dorsalis pedis pulse was absent on the right in 28 patients and on the left in 21 patients. The brachial and radial pulses were absent in one and two patients, respectively (Table 4).

**Table 4:** Absent peripheral arterial pulses by side (n = 50) Data are presented as the number of patients with an absent pulse on each side. The brachial and radial pulses were absent in one and two patients, respectively.

Artery	Absent, right (n)	Absent, left (n)
Dorsalis pedis	28	21
Anterior tibial	25	17
Posterior tibial	17	12
Popliteal	11	6
Femoral	2	0

On ABPI assessment, moderate disease (0.41-0.70) was the most common category (38.0%), followed by mild disease (0.71-0.90; 28.0%) and severe disease (<0.40; 16.0%); 18.0% of patients had a normal or borderline index (Table 5). No patient had a noncompressible vessel (ABPI > 1.40); all measured indices were ≤ 1.29. When the ABPI was interpreted together with clinical findings, PAD was identified in 41 patients (82.0%), whereas nine patients (18.0%) were classified as normal or borderline.

**Table 5:** Distribution of patients by ABPI category (n = 50) Data are presented as n (%). ABPI: ankle-brachial pressure index

ABPI range	Severity	n (%)
1.00-1.29	Normal	5 (10.0)
0.91-0.99	Borderline	4 (8.0)
0.71-0.90	Mild disease	14 (28.0)
0.41-0.70	Moderate disease	19 (38.0)
<0.40	Severe disease	8 (16.0)

**Figure 2:**
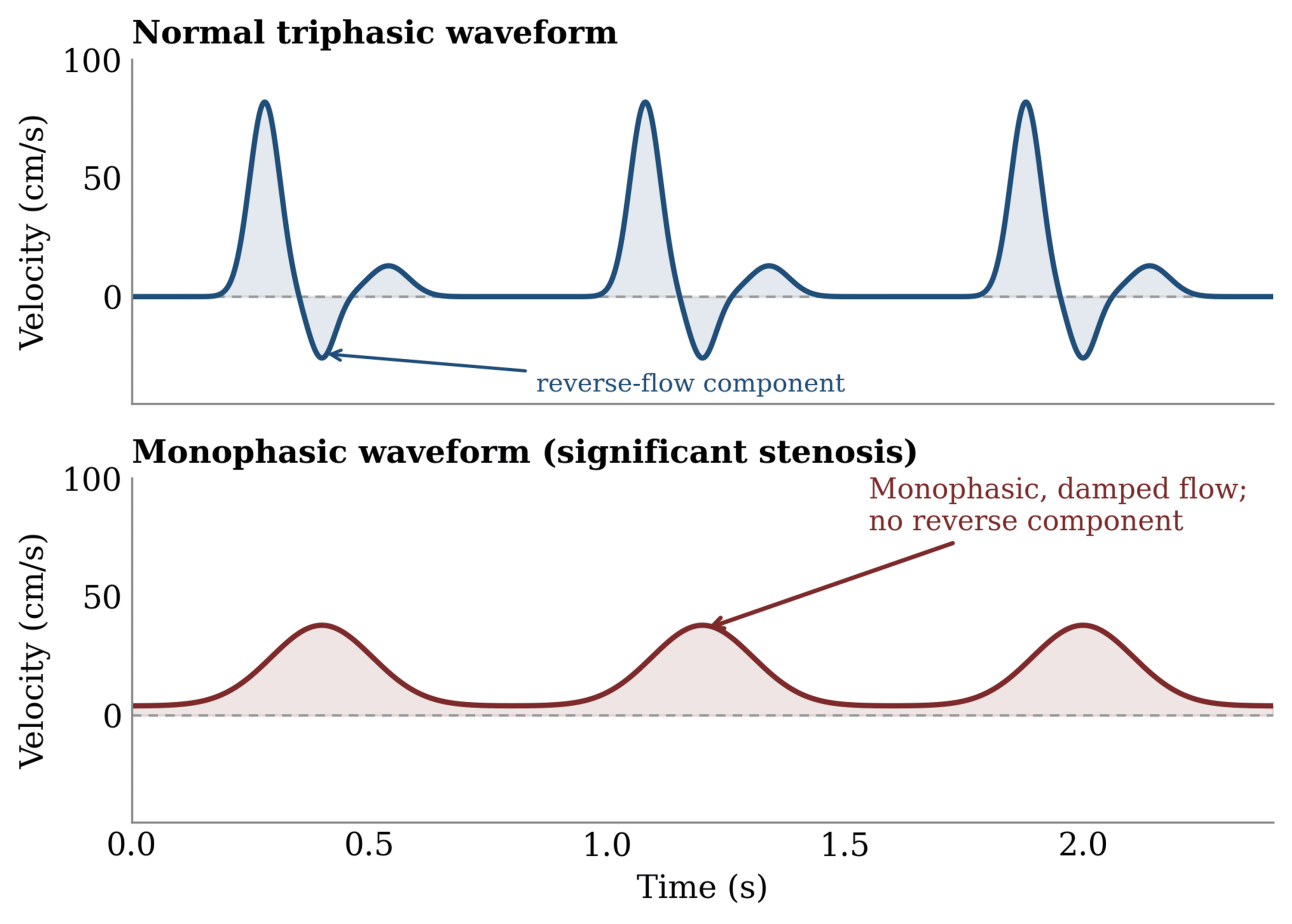
Schematic comparison of normal triphasic and stenotic monophasic lower-limb arterial Doppler waveforms Schematic illustration contrasting a normal triphasic waveform, with a sharp systolic peak, an early diastolic reverse-flow component, and a small late diastolic forward component, with the monophasic, damped, spectrally broadened waveform characteristic of hemodynamically significant stenosis (arrow).

Arterial duplex ultrasonography demonstrated a monophasic flow pattern in 76.0% of patients and stenosis or occlusion in 76.0%, with turbulent flow and spectral broadening in the majority of affected limbs (Table 6). A representative duplex image is shown in Figure 2.

**Table 6:** Arterial duplex ultrasonography findings (n = 50) Data are presented as n (%).

Duplex parameter	n (%)
Flow pattern	
Monophasic	38 (76.0)
Triphasic	12 (24.0)
Spectral window and flow type	
Turbulent	40 (80.0)
Laminar	10 (20.0)
Doppler spectrum	
Spectral broadening	38 (76.0)
High-pulsatility waveform	12 (24.0)
Impression	
Stenosis or occlusion	38 (76.0)
Normal vascular flow	12 (24.0)

The agreement between ABPI plus clinical findings and duplex ultrasonography is shown in Table 7. Of the 38 limbs with stenosis or occlusion on duplex, 37 were also positive by ABPI and clinical assessment, and of the nine patients classified as normal or borderline by ABPI, eight were confirmed to be normal on duplex. Taking the combined ABPI and clinical assessment as the reference, duplex ultrasonography had a sensitivity of 90.2% (95% CI, 77.5-96.1%), a specificity of 88.9% (95% CI, 56.5-98.0%), a positive predictive value of 97.4% (95% CI, 86.5-99.5%), a negative predictive value of 66.7% (95% CI, 39.1-86.2%), and an overall agreement (accuracy) of 90.0% (95% CI, 78.6-95.7%). The association between the two methods was statistically significant (Fisher’s exact test, P < 0.001).

**Table 7:** Agreement between ABPI plus clinical findings and duplex scan (n = 50) Data are presented as the number of patients. The association between the two methods was assessed using Fisher’s exact test (P < 0.001). With ABPI plus clinical assessment taken as the reference, sensitivity was 90.2% (95% CI, 77.5-96.1%), specificity was 88.9% (95% CI, 56.5-98.0%), positive predictive value was 97.4% (95% CI, 86.5-99.5%), negative predictive value was 66.7% (95% CI, 39.1-86.2%), and accuracy was 90.0% (95% CI, 78.6-95.7%). ABPI: ankle-brachial pressure index

Duplex finding	ABPI + clinical positive (n = 41)	ABPI + clinical negative (n = 9)	Total
Stenosis or occlusion	37	1	38
Normal	4	8	12
Total	41	9	50

## Discussion

In this cross-sectional study of 50 patients with lower-limb PAD, the ABPI combined with clinical assessment agreed closely with arterial duplex ultrasonography, achieving a sensitivity of 90.2%, a specificity of 88.9%, and an overall accuracy of 90.0%. These findings support the use of a clinically driven, ABPI-based strategy as an accessible first step in the evaluation of suspected lower-limb arterial disease, with duplex reserved for anatomical localization and treatment planning.

The demographic profile of our cohort is consistent with regional and international experience. The mean age was 53.4 years, with most patients in the fifth and sixth decades, and there was a marked male predominance. A comparable observational study from a tertiary hospital in Bangladesh reported a mean age of 42.8 years, with most patients in the fourth or fifth decade [23]. The somewhat younger age distribution in these South Asian cohorts compared with Western series, in which prevalence rises steeply after 65 years [20,24,25], may reflect lower life expectancy and a heavier burden of unmodified risk factors. Advanced age, cigarette smoking, and diabetes mellitus are consistently identified as the three risk factors most strongly associated with PAD [20].

Smoking was the dominant risk factor in our series, present in 88.0% of patients, in keeping with evidence that tobacco use is the single most important modifiable cause of PAD and that more than 80% to 90% of patients with lower-limb disease are current or former smokers [2,5,15]. Smoking increases the relative risk of PAD severalfold in a dose-dependent manner and is associated with greater disease severity, higher amputation rates, and increased mortality [26,27]. Diabetes mellitus, present in 64.0% of our patients, is recognized to increase the risk of PAD two- to fourfold through endothelial and smooth muscle dysfunction, with risk proportional to the duration of disease; diabetic patients also face a substantially higher likelihood of major amputation [6,28-31]. The prevalence of disease and its detection are further influenced by the diagnostic criteria applied, and a considerable proportion of diabetic patients harbor subclinical disease [32,33].

Hypertension was documented in 58.0% of patients and hyperlipidemia in 10.0%. Elevated blood pressure is correlated with an increased risk of developing PAD and associated coronary and cerebrovascular events, and hypertension has been shown to increase the risk of intermittent claudication severalfold [34]. Dyslipidemia is likewise an established contributor, and correction of lipid abnormalities reduces cardiovascular events; lipid-lowering therapy has a well-documented benefit in reducing rates of myocardial infarction and stroke even without a major reversal of established atherosclerosis [13,35]. The relatively low recorded prevalence of hyperlipidemia in our cohort likely reflects incomplete lipid profiling in a late-presenting population rather than a true absence of risk.

Most patients in our study presented late, with all having tissue loss at the time of assessment and nearly two-thirds seeking care more than six months after symptom onset. Intermittent claudication is the most common early symptom of lower-limb PAD, but many patients are asymptomatic or report atypical symptoms, and the diagnosis is easily overlooked when claudication alone is used as the criterion [14,36]. This pattern of late presentation underscores the value of an objective, inexpensive test such as the ABPI, which can detect disease before critical ischemia develops and also provides prognostic information, as a lower index predicts poorer limb outcomes and survival [16]. In our cohort, the ABPI categorized most patients as having mild to moderate disease, consistent with the spectrum expected in a symptomatic surgical population.

Duplex ultrasonography demonstrated stenosis or occlusion in 76.0% of patients and a predominantly monophasic, turbulent flow pattern, consistent with hemodynamically significant disease. Duplex provides both anatomical and functional information and has high sensitivity and specificity for detecting significant lesions, allowing accurate mapping of disease that can guide the choice between endovascular and surgical intervention [17]. The strong agreement between ABPI plus clinical findings and duplex observed in our study reinforces the principle that combining physiological noninvasive data with imaging yields information vital to therapeutic decision-making, an approach endorsed by contemporary management guidelines [3,37]. Taken together with the broader epidemiology of intermittent claudication in middle-aged populations [38], these findings support routine ABPI screening in patients with suspected PAD, followed by selective duplex imaging.

Our findings have particular relevance for primary care and resource-limited settings. Although the ABPI is inexpensive, portable, and can be performed reliably by trained nonspecialist staff using a handheld Doppler device, it remains underused in primary care, where a large proportion of affected patients are neither identified nor treated [20,21]. In many low- and middle-income settings, access to arterial duplex ultrasonography, computed tomographic angiography, or catheter angiography is limited or concentrated in tertiary centers, and patients, as in our cohort, frequently present late with tissue loss. A pragmatic, stepwise strategy in which clinical assessment and the ABPI are used to identify and risk-stratify patients at the first point of contact, reserving duplex imaging for those who screen positive or who are being considered for revascularization, is therefore attractive and is consistent with the stepwise diagnostic pathway recommended in contemporary guidelines [3,4,37]. The strong agreement between ABPI plus clinical findings and duplex observed here supports the feasibility of such an approach where imaging resources are scarce.

Beyond diagnosis, early identification of PAD creates an opportunity for aggressive secondary prevention because the ABPI is also a marker of systemic atherosclerosis and future cardiovascular risk [16]. Guideline-directed management, including smoking cessation, antiplatelet therapy, statin and other lipid-lowering treatment, blood pressure and glycemic control, and structured exercise, with revascularization reserved for limb-threatening or lifestyle-limiting disease, reduces both limb loss and cardiovascular events [3,4,35,37]. In a population that presents late and carries a high burden of smoking and diabetes, as in the present study, coupling timely ABPI-based diagnosis with systematic risk factor modification is likely to yield substantial benefit at both the individual and population levels.

This study has several limitations that merit consideration. First, the sample was small and recruited by purposive sampling from a single tertiary center, introducing the possibility of selection bias and limiting generalizability. Second, the cohort consisted predominantly of poor, late-presenting patients with established tissue loss, so the spectrum of disease severity was skewed toward advanced disease, and the diagnostic estimates may not apply to asymptomatic screening populations. Third, the cross-sectional design captured a single point in time and did not address longitudinal outcomes. Finally, duplex ultrasonography rather than catheter angiography was used as the comparator, and the diagnostic performance reported reflects agreement between two noninvasive modalities rather than validation against a definitive anatomical reference standard. Larger, multicenter studies with angiographic confirmation and prospective follow-up would help refine these estimates.

## Conclusions

The ABPI, combined with clinical assessment, correlated strongly with arterial duplex ultrasonography in patients with lower-limb PAD, with high sensitivity, specificity, and overall accuracy. Accurate anatomical and physiological assessment can be achieved with modern noninvasive vascular techniques, and the combined use of the ABPI, clinical evaluation, and selective duplex imaging provides the information needed to formulate a rational therapeutic plan. Health care providers should make every effort to detect PAD early, assess associated cardiovascular risk factors, and institute appropriate long-term management, particularly in resource-limited settings where late presentation is common.
